# Effectiveness of exercise interventions on fall prevention in ambulatory community-dwelling older adults: a systematic review with narrative synthesis

**DOI:** 10.3389/fpubh.2023.1209319

**Published:** 2023-08-03

**Authors:** Munseef Sadaqa, Zsanett Németh, Alexandra Makai, Viktória Prémusz, Márta Hock

**Affiliations:** ^1^Faculty of Health Sciences, Doctoral School of Health Sciences, University of Pécs, Pécs, Hungary; ^2^Faculty of Health Sciences, Institute of Physiotherapy and Sport Science, University of Pécs, Pécs, Hungary; ^3^Physical Activity Research Group, Szentágothai Research Centre, Pécs, Hungary

**Keywords:** fall, older adults, exercise, strength, balance, multi-component

## Abstract

**Objective:**

To present a systematic review of randomized controlled trials which summarizes the effects of community-based resistance, balance, and multi-component exercise interventions on the parameters of functional ability (e.g., lower extremities muscle strength, balance performance and mobility).

**Methods:**

This PROSPERO-registered systematic review (registration no. CRD42023434808) followed the PRISMA guidelines. Literature search was conducted in Cochrane, Embase, Ovid Medline, PEDro, Pubmed, Science Direct, Scopus and Web of Science. We included RCTs that investigated the following interventions: lower extremity strengthening, balance and multi-component exercise interventions on ambulatory community-dwelling adults aged ≥65 years.

**Results:**

Lower extremity strengthening exercises revealed significant effects on the strength of lower extremity, balance outcomes and mobility. Balance exercises reduce the rate of injurious falls, improve static, dynamic and reactive balance, lower extremity strength as well as mobility. Multi-component exercise training reduces medically-attended injurious falls and fallers, incidence of falls, fall-related emergency department visits as well as improves mobility, balance, and lower extremity strength.

**Conclusion:**

Physical exercises are effective in improving the components of balance, lower extremity strength, mobility, and reducing falls and fall-related injuries. Further research on fall prevention in low-income countries as well as for older adults in vulnerable context is needed.

## 1. Introduction

One-third of older adults aged 65 years or over fall each year, and 50% of them fall repeatedly (1). According to the World Health Organization (WHO) (2), falls are the second leading cause of unintentional injury deaths globally, and the literature shows that 40% of community-dwelling older adults who are over 65 years experience fall accidents annually (3). In Europe, every year approximately 36,000 older adults (65 years and above) die from falls (data 2010–2012). Of these, 88% of cases are related to people aged 75 years or older and 59% of cases are related to women (4).

Physical inactivity and deficits in functional ability (e.g., reduced lower limbs muscular strength and impaired balance ability) have been identified as independent contributors to falls and fall-related injuries (e.g., head injuries and hip fractures) among older adults (5–8). Even though they are critical risk factors for older adults, they are modifiable and preventable through exercises (7, 9, 10). Falls also have economic burdens such as medications, hospital admissions, and extended rehabilitation services (2, 11).

Exercise training is broadly recognized as an appropriate intervention strategy for improving functional performance in older adults, reducing the aforementioned risk factors, and consequently minimizing the risk of falling (12–14). Additionally, the reduction of physical inactivity has been shown to have positive economic impacts on national, social, and individual levels (15).

Cognitive decline also occurs during normal aging (16), which is considered a significant factor in increased functional dependence and fewer activities of daily living among older adults (17). According to previous studies, older adults who exercised three times a week for 6 months showed improvements in global and executive cognitive functions as well as a general deceleration of the aging process (18, 19).

Accordingly, the WHO recommends that older adults aged over 65 years adopt an active lifestyle that ensures the performance of physical activity of moderate intensity for a minimum of 150 min weekly (20, 21), as well as engage in fall prevention exercises (22). This moderate amount of physical activity has been shown to reduce the risk factors associated with falls by up to 50% by reducing functional limitation in older persons, and eventually reducing the risk of falls (13).

Several studies (23–28) have concluded that strength, balance, and aerobic-based training is effective in reducing falls among older adults in general and up to 50% in community-dwelling older adults specifically, with gains that may last as the training is maintained (29). In their clinical guideline for the prevention of falls, the American Geriatrics Society and British Geriatrics Society Clinical Practice recommend that the prevention of fall risk factors should include gait training, resistance, and balance exercises (30).

Studies on resistance exercises reported their effectiveness in mitigating reduced muscle strength, and that these types of exercises are considered an essential element of a multi-component exercise fall prevention strategy (31, 32). Furthermore, there is a considerable body of literature on balance exercises and their effectiveness in reducing the risk of falling among community-dwelling older adults (23, 33–36).

Previous systematic reviews and meta-analyses explored the effects of exercise on fall prevention among older people. However, these reviews and meta-analyses were conducted in a younger age group (60 years and over) (23, 27) compared with our present review and in participants with Parkinson’s disease, stroke, and cognitive impairment (26) who had mixed living settings (living in the community, nursing homes, and higher dependency places of residence) (35, 37). They concluded that exercises as a stand-alone intervention can significantly reduce the rate of falls (23, 26, 27, 35, 37) and the risk of falling (23, 27).

To the best of our knowledge, despite the current interest in the topic, there is a lack of systematic reviews on fall prevention strategies that address the effectiveness of exercise training, specifically on ambulatory community-dwelling older adults aged 65 years or older. Therefore, our current review aims to present a systematic review of randomized controlled trials (RCTs) that summarizes the effects of resistance, balance, and multi-component exercise interventions. These interventions consist mainly of combined resistance and balance exercises in addition to one or more types of exercises, namely, aerobic, walking, and weight-bearing, on the parameters of functional ability (e.g., lower extremities muscle strength, balance performance, and mobility), which accordingly prevent falls in ambulatory community-dwelling adults aged 65 years and over.

## 2. Methods

The current systematic review is presented in accordance with the guidelines of PRISMA 2020 (Preferred Reporting Items for Systematic Reviews and Meta-Analyses literature search extension) (38). The review was registered on PROSPERO with the following registration no. CRD42023434808.

### 2.1. Data sources and search strategies

Two consecutive searches were conducted by two researchers (MS and ZN) independently. The initial search was limited to studies in English published from 1 January 2015 to 30 December 2020, then the search was conducted again on 7 August 2021 for publications published from 1 January 2021 to 7 August 2021 in the following electronic databases: Cochrane Central Register of Controlled Trials, Embase, Ovid Medline, PEDro, Pubmed, Science Direct, Scopus, and Web of Science. The search was supplemented by a manual review of reference lists from included primary studies and review articles to find additional studies on the subject (Table 1).

**Table 1:** Search strategy.

Database	Search strategy
Cochrane Library, Embase, Pubmed, Science Direct, Scopus, and Web of Science	((senior* OR elder* OR old) AND (exercis* OR train*) AND (effect* OR benefit*) AND (lower limb* OR lower extremit*) AND (fall*))
PEDro	Abstract & Title: fall* exercis* Subdiscipline: gerontology Method: clinical trial/practice guideline Published Since: 2015 When Searching: Match all search terms (AND)
Ovid Medline	“Aged, 80 and over”/ or Aged/ Limit 1 to (abstract and English language and yr. = “2015-Current and (“all aged (65 and over)”) and (clinical trial, all or clinical trial or randomized controlled trial)) Accidental Falls/pc [Prevention & Control] Limit 1 to (abstract and English language and yr. = “2015-Current” and (“all aged (65 and over)”) and (clinical trial, all or clinical trial or randomized controlled trial)) Polymetric Exercise/ or Exercise Therapy/ or Exercise/ Limit 1 to (abstract and English language and yr. = “2015-Current and (“all aged (65 and over)”) and (clinical trial, all or clinical trial or randomized controlled trial)) 2 and 4 and 6

All research papers were first retrieved, and Zotero 6.0.26 (a reference manager created at the Roy Rosenzweig Center for History and New Media at George Mason University, Virginia, United States) was utilized to remove duplicates. Next, titles and abstracts were screened independently by two researchers (MS and ZN), and finally, the full texts of the remaining studies were assessed to determine eligibility. Disagreements between the two researchers were resolved through discussion and mutual consent, or by a third assessor (MH). After the abstract and full-text analysis, the Cohen’s Kappa-coefficient (K score) was calculated to weigh the level of agreement between the two reviewers.

### 2.2. Eligibility criteria

Based on PICOTS, studies were included if they were written in English, were RCTs on community-dwelling adults aged ≥65 years, and included the following interventions: lower limb strengthening exercises, balance exercises, and multi-component exercise interventions that consist of resistance and balance exercises in addition to one or more types of exercises, namely, aerobic, walking, and weight-bearing. Studies were excluded if (I) they involved residents of nursing homes and inpatients, (II) involved participants with a diagnosis of multiple sclerosis, cancer, Parkinson’s or Alzheimer’s disease, stroke, recent fractures, vision or cognitive impairments, or other chronic conditions associated with aging, (III) they were on non-human participants, (IV) they were based on interventions of video games, web-based programs, Tai Chi, Otago exercise program, multifactorial components that combine exercises and non-exercise interventions, yoga, dance therapy, or water-based exercise program, and (V) were non-RCTs, cohort studies, cross-sectional studies, reviews, conference abstract/papers, surveys, opinion pieces, commentaries, books, periodicals, editorials, case studies, non-peer-reviewed articles, masters dissertations, and doctoral theses.

### 2.3. Data extraction

Two reviewers independently extracted the following data from each study: author, publication year, study title, aim and design, number and demographic data of participants, the type of intervention such as lower limb strengthening exercise training, balance exercise training, and multi-component exercise training, duration, frequency, and settings of intervention, equipment used, outcome measures, results, and limitations.

### 2.4. Risk of bias assessment

One review author (MS) carried out the risk of bias assessment using Cochrane’s Risk of bias tool among six domains: random sequence generation, allocation concealment, blinding of participants and personnel, blinding of outcome assessment, incomplete outcome data, and selective reporting (38).

## 3. Results

### 3.1. Study selection

**Figure 1:**
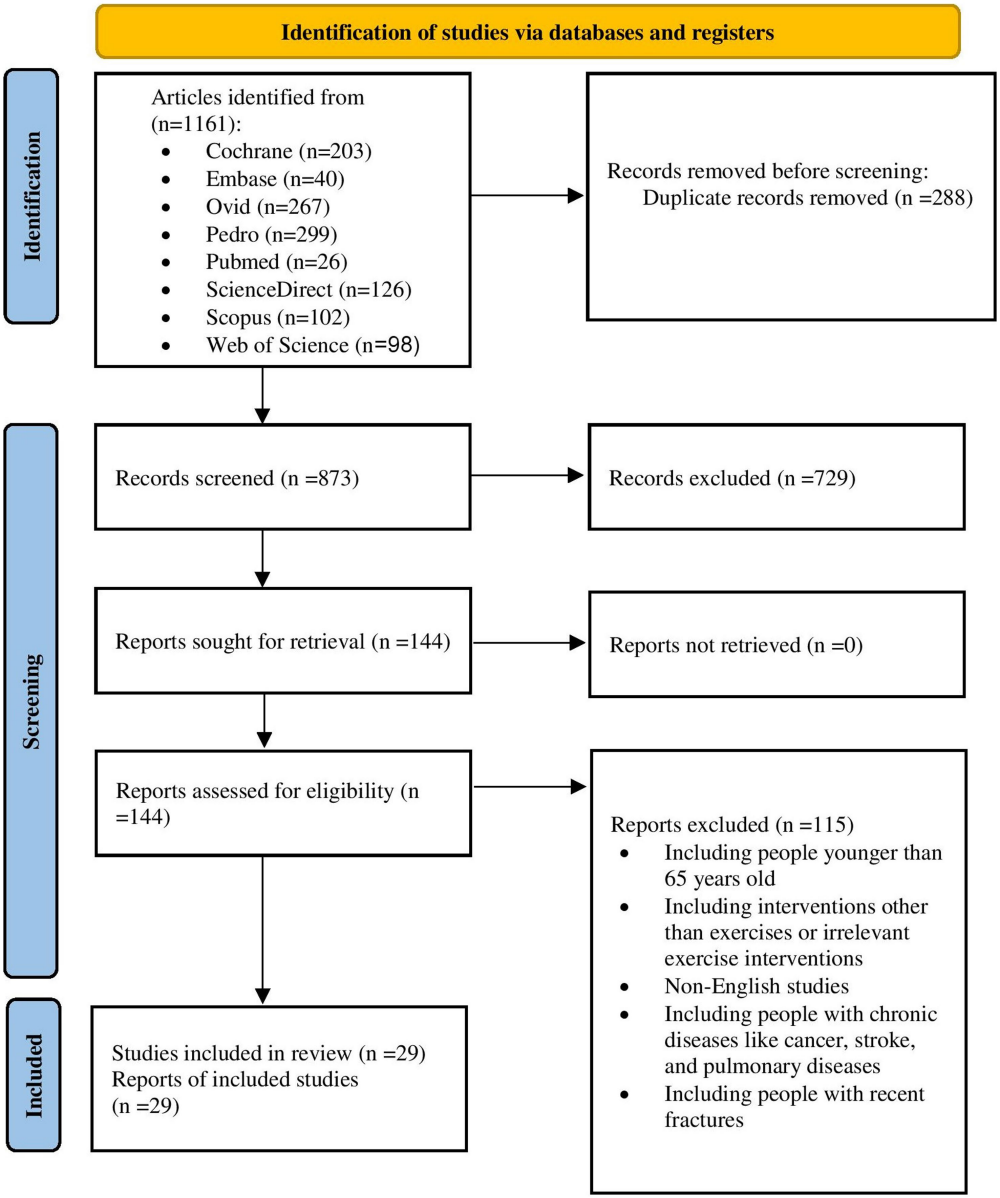
Flow of the studies screened.

From 1,161 yielded studies, 288 duplicates were excluded. Then, following title and abstract screening, another 729 were excluded. The remaining studies were assessed against the inclusion and exclusion criteria and 29 studies in total were eligible to be included in the systematic review (Figure 1). To measure the inter-rater reliability between the two reviewers, a K score was calculated at abstract level (0.82) and at full-text level (0.86), which showed a high level of agreement. The included studies involved 4,330 participants, of which 4,121 adhered to the end of the studies and were analyzed for the outcome measures. The participants’ average age ranged from 66.4 to 82.4 years, and all were community-dwelling older adults. The 29 included studies were only RCTs.

### 3.2. Setting and training equipment

Interventions were mainly conducted in gyms, exercise halls, community facilities (5, 32, 39–41), combined gyms, home-based locations (42–45), and laboratories (46, 47). Some studies did not report the intervention setting, however, a gym/community setting was likely from the description of interventions (48–65).

A variety of training equipment was used: resistance training machines (32, 45, 46, 49, 52, 53, 55, 61), recumbent trainer, seated stationary cycle ergometer, treadmills, climber, bike recline (32, 47, 49, 52), weighted equipment such as cuff and vest weights (5, 32, 39–41, 45, 50, 55, 63, 65), elastic bands/tubing (39–41, 53, 56, 58, 59), free weights (45, 52), body weight (5, 44, 48), balls (39, 42, 44, 50, 51), step-boards (5, 39, 45), chairs (5, 50), equipment used for balance training such as tripping boards, slipping tiles, balance foams, wedged soft mat, soft pads, non-inverted BOSU^®^ balls, inverted BOSU^®^ balls, balance cushions, balance beams, semicircular blocks, Posturomed^®^, wobble boards, and inflatable discs (39–41, 43, 48, 52, 53, 57). One study reported that they used small, low-cost equipment as well (e.g., towels and bottles) (44), whereas five studies did not clearly report any equipment used, however, they could have merely used body weight for exercises (54, 60, 62, 64, 65).

### 3.3. Dosage of exercise programs

The duration of exercise interventions ranged from 15 min (44) to 90 min (53, 64, 65) per session, the frequency of interventions ranged from once a day (43) to five times a week (62), and the duration of the exercise programs ranged from 1 week (47, 57) to 2 years (5, 42).

### 3.4. Results according to intervention

#### 3.4.1. Lower limb strengthening exercise training

Seven of the included studies contained lower limb strengthening exercises as the only intervention or one of a multi-component intervention. They used different modalities and showed significant improvements in lower extremities strength (using dynamometer, five times sit to stand test, and chair rise test), balance (activities specific balance confidence (ABC) scale, berg balance scale, one leg standing test, Y-balance test, and functional reach test), mobility (6-minute walk test, timed up and go test (TUGT), and 30-second chair stand test), days survived without a fall or near-fall, gait (functional gait assessment) (32, 46, 52–54, 56, 59). One study did not show significant change in static balance (single leg stance assessment with eyes closed on the dominant and single leg stance assessment with eyes closed on the non-dominant) or eccentric strength (maximal eccentric strength) (46). Another study did not show significant improvement in TUGT, except when exercise was combined with neuromuscular electrical stimulation (54). Table 2 shows a complete summary of the key data extracted from the studies on strengthening exercise interventions.

**Table 2:** A complete summary of the key data extracted from studies of strengthening exercise intervention.

References	Participants	Interventions modality	Duration	Outcomes	Summary of results
Eckardt (52)	83 (48 women, 35 men) Age 65–80	M-SRT M-URT: followed a similar training program with M-SRT, but with additional unstable devices placed between the participant and the exercise machine or floor, respectively, F-URT using dumbbells	10 weeks	Isometric leg extension strength CRT: muscle power TUGT: proactive balance FRT: proactive balance PRT: reactive balance	Significant improvements for all outcome measures in all intervention groups
Hamed et al. (53)	63 (women and men) Age 65–80	Lower extremity muscle strength using resistance training machines and Thera^®^ bands with different stiffness Perturbation-based dynamic stability on different unstable undergrounds	14 weeks	LoS MoS at release MoS at touchdown BoS at touchdown Maximum voluntary isometric knee extension moment Maximum voluntary isometric ankle plantar flexion moment	- Significant improvements for MoS and knee and ankle momentum in muscle strength group -Significant improvement for LoS, MoS and ankle momentum muscle strength group
Jang and Park (54)	30 (women only) Age ≥ 65	Lower limb muscle strengthening	4 weeks	STST: functional lower limb muscle strength, balance control ability, and fall risk TUGT: functional mobility and dynamic balance ability OLST: static balance YBT: dynamic balance	- Significant differences in STST, OLST and YBT - No significant difference with TUGT
Johnson et al. (46)	30 (16 women, 14 men) Age 68.2 ± 3.7	Eccentric exercise on a specialized eccentric exercise machine	8 weeks	30CST: mobility BBS: holistic assessment of balance SLS-EC:D and SLS-EC:ND: balance FGA: gait TUGT: mobility MES: Maximal eccentric strength	- Significant improvement in for 30SCST, BBS, FGA and TUGT - No significant improvement in SLS-EC:D, SLS-EC:ND and MES
LaStayo et al. (32)	134 (47 men, 87 women) Age ≥ 65	Resistance exercise via negative, eccentrically-induced work (RENEW) Traditional (TRAD) resistance exercise	3 months	6MWT: mobility ABC Scale: self-reported level of balance confidence Leg extensor muscle power Fall and near-fall events (days survived without a fall or near-fall)	-No significant difference between RENEW and TRAD in 6 MWT -No significant difference between RENEW and TRAD in ABC Scale -No significant difference between RENEW and TRAD in leg extensor muscle power -No significant difference between RENEW and TRAD in the number of days survived without fall
Lee and Lee (56)	30 (women only) Age ≥ 65	Closed and open kinetic chain exercises using elastic bands	4 weeks	OLST: static balance FRT: dynamic balance	- Significant difference in OLST and FRT - No significant difference between the two groups
Pourtaghi et al. (59)	70 (48 women, 22 men) Age ≥ 65	Resistance training using Thera-Band	6 weeks	Lower extremity strength (leg press, twin press, front thigh press, back thigh press, and posterior leg press) using a dynamometer	- Significant ↑ in both lower extremities strength

30SCST, 30-Second Chair Stand Test; 6 MWT, 6-Minute Walk Test; ABC, Activities Specific Balance Confidence; BBS, Berg Balance Scale; BoS, Base of support; CRT, chair Rise Test; F-URT, Free-weight Unstable Resistance Training; FGA, Functional Gait Assessment; FRT, Functional Reach Test; LoS, Limits of Stability; M-SRT, Machine-based Stable Resistance Training; M-URT, Machine-based Unstable Resistance Training; MES, Maximal Eccentric Strength; MoS, Margin of Stability; OLST, One Leg Standing Test; PRT, Push and Release Test; SLS-EC,D, Single Leg Stance assessment with Eyes Closed on the Dominant; SLS-EC,ND, Single Leg Stance assessment with Eyes Closed on the Non-Dominant; STST, Sit To Stand Test; TUGT, Timed Up and Go Test; YBT, Y-Balance Test.

#### 3.4.2. Balance exercise training

Ten studies compared balance exercise interventions to non-active controls (usual daily activities and social programs), and those that used different balance interventions methods showed significant improvements in muscle strength [chair standing test (CST), four square step test (FSST), and 2-min step test (2MST)], balance [single leg stance test (SLST), one leg standing test, tandem stance test, ABC scale, modified Clinical Test of Sensory Organization and Balance, local dynamic stability, and balance recovery], gait and mobility (TUGT, 6-min walk test, five times chair stand test, and functional reach test), lower rate of falls, reduction in the rate ratio of all falls, and slip falls (42, 43, 47, 50, 53, 57, 60, 62, 64, 65).

One RCT compared an enhanced balance program with a standard balance program on sit-to-stand test repetitions. The study revealed no significant difference between the two groups in sit-to-stand test repetitions. There was a statistically significant improvement in sit-to-stand test repetitions from pre- to post-intervention for both standard balance and enhanced balance exercise groups (39).

A study in this review scrutinized the effects of an 8-week square-step exercise in older fallers. The composite balance score as a marker of postural control was significantly higher in the square-step exercise group compared with the vestibular exercise group. Adaptation test was utilized to assess the ability to control motor reactions after an unexpected change in position (dynamic balance). Square step exercise did not show significant results compared with vestibular exercise neither in toes up nor in toes down (48). Table 3 shows a complete summary of the key data extracted from the studies on balance exercise interventions.

**Table 3:** A complete summary of the key data extracted from studies of balance exercise intervention.

References	Participants	Interventions modality	Duration	Outcomes	Summary of results
Arghavani et al. (50)	60 (men only) Age 65–80	Perturbation training: catching, throwing, dribbling and passing balls while sitting and standing on stable and unstable surfaces Balance training: standing balance, walking balance and strength training	8 weeks	ABC: balance confidence	- Significant ↑ in Balance confidence in both training programs
Arnold et al. (39)	28 (women and men) Age ≥ 65	Standard balance: progressive strengthening for upper and lower body, 10 to 15 min functional balance exercises Enhanced balance: same as above, with the addition of a progressive core stabilization	9 weeks	STST: balance and postural control MoS: balance and postural control	- Significant improvement in STST for both interventions - No significant improvement in MoS for both interventions
El-Khoury et al. (42)	706 (women only) Age 75–85	Progressive balance training: postural stability, muscle extensibility, joint flexibility, balance, reaction time, coordination, muscle strength and internal sense of spatial orientation	2 years	Rate of serious injurious falls Rate of moderate injurious falls Rate of all falls TUGT: balance, gait, and motor function 6MWT: balance, gait, and motor function 5TSST: balance, gait, and motor function SLST: balance, gait, and motor function	- Significant improvements in all balance and gait tests at one and 2 years
Hamed et al. (53)	63 (women and men) Age 65–80	Lower extremity muscle strength using resistance training machines and Thera^®^ bands with different stiffness Perturbation-based dynamic stability on different unstable undergrounds	14 weeks	LoS MoS at release MoS at touchdown BoS at touchdown Maximum voluntary isometric knee extension moment Maximum voluntary isometric ankle plantar flexion moment	- Significant improvements for MoS and knee and ankle momentum in muscle strength group -Significant improvement for LoS, MoS and ankle momentum muscle strength group
Hirase et al. (43)	93 (65 women, 28 men) Age > 65	Balance training program on a foam rubber pad or on a stable flat surface	4 months	OLST: static balance CST: lower-extremity strength TUGT: dynamic balance TST: static balance	- Significant ↑time (Improvement) in OLST on foam rubber, but not significant in stable surfaces - Significant ↓time (Improvement) in CST and TUGT for both intervention groups - Significant ↑time (Improvement) in TST for both intervention groups
Kocaman et al. (48)	42 (women and men) Age > 65	Posturography balance exercise Square step exercise	8 weeks	Composite balance score: postural control ADT: dynamic balance	- Significant improvements in composite balance score and ADT for both interventions
Okubo et al. (57)	44 (25 women, 19 men) Age 65–90	Reactive balance training	1 week	Fall incidence MoS (cm) Trunk sway range (degree): (postural stability during recovery)	- Significant ↓ in all falls and slip falls, but not significant in trip falls
Rieger et al. (47)	30 (women and men) Age > 65	Treadmill training session with 16 anterior–posterior perturbations	1 week	Local divergence exponent for anterior–posterior: local dynamic stability Local divergence exponent for medio-lateral: local dynamic stability	- Significant differences for all outcome measures in the intervention group
Sadeghi et al. (60)	64 (men only) Age ≥ 65	Balance Training: single-leg stance with eyes open and closed, standing on heels or toes, tandem and semitandem foot stance, tandem walking, walking backward and forward, and weight shifting.	8 weeks	Isokinetic muscle strength of lower limbs was quantified by means of the Biodex Isokinetic Dynamometer SLST: balance TST: balance TUGT: functional mobility 10mWT: functional mobility	- No significant difference in muscle strength - Significant ↑ time (Improvement) in SLST -Significant ↑ time (Improvement) in TST - Significant ↓time (Improvement) in TUGT (improvement) - Significant ↓ time (Improvement) in 10mWT
Sitthiracha et al. (62)	60 (53 women, 7 men) Age 65 to 75	Progressive step marching exercise: balance training	8 weeks	TUGT: balance ability OLST: balance ability 5TSST: lower limb muscle strength	- Significant ↓ time (Improvement) in TUGT - No significant improvement in OLST - Significant ↓ time (Improvement) in FTSST - Significant ↑ steps (Improvement) in 2MST
Zhao et al. (64)	61 (42 women, 19 men) Age 65–74	Exercise for Balance Improvement Program, ExBP	16 weeks	FRT: postural control ability (dynamic balance test) m-CTSIB: static balance with compromised vision, vestibular, and somatosensation	- Significant improvement in FRT and m-CTSIB
Zhao et al. (65)	61 (42 women, 19 men) Age 65–74	Exercise for Balance Improvement Program	16 weeks	30SCST: lower extremity muscle strength UG: agility and dynamic balance	- Significant ↑ repetition (Improvement) in 30SCST - Significant ↓ time (Improvement) in UG

5TSST, Five Times Sit to Stand Test; 10mWT, 10-Meter Walk Test; 30SCST, 30-Second Chair Stand Test; 6 MWT, 6-Minute Walk Test; ABC, Activities Specific Balance Confidence; ADT, Adaptation Test; BoS, Base of support; CST, Chair Standing Test; FRT, Functional Reach Test; LoS, Limits of Stability; m-CTSIB, Modified Clinical Test of Sensory Organization and Balance; MoS, Margin of Stability; OLST, One Leg Standing Test; SLST, Single Leg Stance Test; STST, Sit To Stand Test; TST, Tandem Stance Tet; TUGT, Timed Up and Go Test, UG, 8 ft Up-and-Go Test.

#### 3.4.3. Multi-component exercise training

Seven studies that compared multi-component exercise interventions to non-active controls showed significant improvements in lower extremities strength (isometric leg extension strength, CST, and knee extensors strength), static balance (backward walking, Romberg test, and postural sway), proactive balance (functional reach test), reactive balance, and mobility, as well as showed lower medically-attended injurious falls and a decrease in the frequency of fallers (5, 44, 45, 49, 51, 55, 58).

An RCT by Li and colleagues aimed to assess the effectiveness of multimodal exercise (MME) training consisting of balance, aerobics, strength, and flexibility activities relative to stretching exercises (SE) in reducing the incidence of falls in older adults. MME showed a significantly lower incidence of falls (16 per 100 person-months) compared with SE (27 per 100 person-months). For total walking duration in instrumented-TUGT and short physical performance battery, which measured repeated chair stands, three increasingly challenging standing balance tasks, and a 4-m speed walk, the MME group performed significantly better than those in the SE group (40).

Another RCT aimed to assess the longer-term effectiveness of multimodal exercise (MME) training consisting of balance, aerobics, strength, and flexibility activities relative to therapeutic Tai Ji Quan: Moving for Better Balance (TJQMBB) and SE in decreasing injurious falls among older adults at high risk of falling. For moderate and serious injurious falls, MME demonstrated significantly lower incidence compared with SE (41). One RCT examined the effect of an 8-week-long two-exercise routine: 1. Strength and core stability training, which consisted of core stability (planks, climb exercise, and supine bridge) and strengthening exercises and 2. Strength and aerobic training, which consisted of aerobic exercises (bike recline and treadmill) and strengthening exercises. Lower limb strength was assessed by a 30-s chair stand test, aerobic ability by 2MST, and finally static balance by SLST. In the strength and core stability training group, tests revealed significant differences in the 30-s chair stand test, in the left SLST, in the right SLST, and finally in 2MST (61). In the strength and aerobic training group, there was only a significant difference between pre- and post-tests for 2MST. Others examined the effectiveness of an 8-week complex exercise program on walking ability and fall efficacy compared to a general exercise program. The complex exercise program consisted of resistance exercises to strengthen trunk, stretching exercises to increase flexibility, and aerobic exercises. FSST was used to assess complex walking ability. Figure-of-8 walking test (F8WT) is another test that is used to measure curved walking ability. During F8WT, the subject is asked to walk a “figure of 8” pattern around cones, the number of steps and time to accomplish the test are considered. For FSST and F8WT, in the complex exercise program and general exercise program there was significant difference between pre and post-tests. In addition, there was a significant difference between the complex exercise program and general exercise program for FSST, but not for F8WT. The results signify complex exercise program effectiveness in improving balance skills while changing direction and following a curved line (63). Table 4 shows a complete summary of the key data extracted from the studies on multi-component exercise interventions.

**Table 4:** A complete summary of the key data extracted from studies of multi-component exercise intervention.

References	Participants	Interventions modality	Duration	Outcomes	Summary of results
Ansai et al. (49)	69 (47 women, 22 men) Age > 80	Multicomponent training group: warm-up, aerobic, strength, balance, and cool-down exercises Resistance training group: strength exercises using six adapted machines	16 weeks	5TSST: muscle strength of the lower limbs OLST: balance TT: balance TUGT-M: dual task Fall incidence	- Significant improvements for all outcome measures
Chittrakul et al. (51)	72 (women and men) Age ≥ 65	MPE program (proprioception training, muscle strength training, reaction time exercise training with auditory cues, and postural balance training)	12 weeks	Knee extension strength Postural sway	- Significant improvements for knee extension strength and Postural sway
Karinkanta et al. (55)	149 (women only) Age 70–78	Combined resistance and balance-jumping training (COMB) Resistance training Balance-jumping training	12 months	Rate of injured fallers during the 5-year follow-up period Rate of injurious falls Fall-induced fractures	- ↓ rate of injured fallers for COMB - ↓ risk for injurious falls for COMB - ↓ risk for fractures COMB
Lacroix et al. (44)	66 (41 women, 25 men) Age 65–80	Combined balance and strength training: using participants’ own body weight or with the help of small, low-cost equipment (e.g., towels, bottles, balls).	12 weeks	ROMT: static steady-state balance Stride velocity: dynamic steady-state balance Stride length: dynamic steady-state balance FRT: proactive balance TUGT: proactive balance Mediolateral perturbation impulse: reactive balance PRT: reactive balance CST: lower extremity muscle power SADT: lower extremity muscle power	- Significant (Improvement) in ROMT, stride velocity and length, for supervised group only - Significant (Improvement) in FRT, TUGT, PRT, CST and SADT for both supervised and unsupervised groups - Significant (Improvement) in mediolateral perturbation for unsupervised group only
Li et al. (40)	670 (436 women, 234 men) Age ≥ 70	Multimodal exercise: balance, aerobics, strength, and flexibility activities	24 weeks	Incidence of falls iTUG: walking duration (in seconds) and 3 subdomain timed-based activities; sit-to-stand, turning, and turn and stand-to-sit—during a 14-m walk at normal pace SPPB: repeated chair stands (strength), 3 increasingly challenging standing balance tasks, and a 4-m speed walk.	- Significant ↓incidence of falls - Significant ↓ time (Improvement) in iTUGT - Significant ↑score (Improvement) in SPPB
Li et al. (41)	670 (436 women, 234 men) Age ≥ 70	Multimodal exercise: balance, aerobics, strength, and flexibility activities	24 weeks	Number of moderate injurious falls during the 12 months Number of serious injurious falls during the 12 months Number of fall-related emergency department visits Number of fall-related hospitalizations	- Significant ↓ injurious falls - Significant ↓ serious injurious falls - Significant ↓ fall-related emergency department visits - No significant ↓ in fall-related hospitalizations
Park et al. (58)	22 (women only) Age ≥ 65	Walking, senior-robic exercises, and muscle strengthening exercises for the lower extremities using elastic bands	12 weeks	Muscle activation of the right and left rectus femoris Muscle activation of the right and left biceps femoris 5.Muscle activation of the right and left tibialis anterior Muscle activation of the right and left gastrocnemius	- Significant ↑ in muscles activation
Patil et al. (45)	409 (women only) Age 70–80	Multimodal exercise (balance challenging, weight bearing, strengthening, agility, and functional exercises)	24 months	Maximal isometric leg extensor strength (using a strain gauge dynamometer) SPPB: mobility 4-m fast walking speed: mobility TUGT: mobility 6.1-m backward walking test: dynamic balance Rate of falls During the 24-Month intervention No. of fallers with different consequences during the 24 Month intervention	- Significant improvement in isometric leg strength and fast walking speed - No significant differences between groups in TUGT - Significant probability of completing backward walking -No significant difference between groups in the total falls incidence rate ratio or falls with any reported injury - Significant ↓medically attended fall injuries and medically attended fallers -No significant difference in the number of fallers who were injured
Sannicandro (61)	65 (34 women, 31 men) Age ≥ 65	Strength and core stability group (SCG) Strength and aerobic group (SAG)	8 weeks	CST: lower limb strength SLST: static balance	- Significant difference (Improvement) in CST and SLST for SCG - No significant differences in CST and SLST for SAG
Song and Kim (63)	40 (women and men) Age 68.81 ± 3.48	Complex exercise program CEP (resistance and aerobic exercises) General exercise program GEP	8 weeks	FSST: complex walking ability F8WT: walking skills in older adult persons with mobility impairments FES: degree of self-confidence regarding falling	- Significant improvements in FSST, F8WT and FES for both interventions
Uusi-Rasi et al. (5)	409 (women only) Age 70–80	Strength, balance, agility, and mobility	2 years	IRR for all falls SPPB: static balance, 4 m normal walking speed and five-time chair stand tests (strength), Timed up and go (TUGT) Dynamic balance isometric leg-extensor strength IRR minor falls IRR for medically attended injurious falls HR for all fallers HR for medically attended injurious fallers	- No differences in all falls or minor injurious falls IRR. - ↓ medically attended injurious falls. - ↓ medically attended injured fallers. - TUGT time remained unchanged at follow up tests. - Significant ↑of maximal isometric leg extensor strength

5TSST, Five Times Sit to Stand Test; CST, Chair Standing Test; F8WT, Figure-of-8 Walking Test; FES, Falls Efficacy Scale; FRT, Functional Reach Test; FSST, Four Square Step Test; HR, Hazard Ratio; IRR, Incidence Rate Ratio; iTUGT, Instrumented Timed Up and Go Test; OLST, One Leg Standing Test; PRT, Push and Release Test; ROMT, Romberg Test; SADT, Stair Ascent and Descent Test; SLST, Single Leg Stance Test; SPPB, Short Physical Performance Battery; TT, Tandem Test; TUGT, Timed Up and Go Test.

#### 3.4.4. Risk of bias

The result of the risk of bias summary and risk of bias graph are available in Supplementary Figures S1, S2, respectively. For random sequence generation and allocation concealment, more than 50% of the studies showed unclear risks as they were not clearly described. For blinding of participants and personnel and blinding of outcome assessment, slightly over 50% of the studies showed low risk. Almost 75% of the studies had low risk in the domain of incomplete outcomes. All studies except one demonstrated a low risk for selective reporting.

## 4. Discussion

This systematic review aimed to present a summary of the effectiveness of resistance, balance, and multi-component exercise interventions, which consist mainly of combined resistance and balance exercises in addition to one or more types of exercises, namely, aerobic, walking, and weight-bearing, on the parameters of functional ability (e.g., lower extremities muscle strength, balance performance, and mobility), which accordingly prevent falls in ambulatory community-dwelling adults aged 65 years or above.

Although poor muscle strength is an established risk factor for falls (66), it has been found that the inclusion of strength training had no substantial effect on falls (23, 35, 37) when the person has sufficient strength to avoid falling (35). Moreover, strength training as a single intervention showed no evidence to be effective in fall prevention (23). However, strength training is likely to offer older adults with longer-term fall prevention and other health gains (37, 67–69), and these types of exercises are recommended to be included in addition to other training (i.e., balance training) (37). To be effective, strength training should provide a certain amount of resistance in an exercise and maximally 10–15 repetitions should be completed before reaching muscle fatigue (67). A systematic review concluded that there is a positive association between the type of resistance exercise and the effect on strength gain and that resistance exercises also help in preserving functional independence and quality of life in older adults (70). In a previous study, eccentric strengthening exercise, when compared to traditional strengthening exercise, yielded greater improvements in muscle strength, balance, and mobility (71). Concerning the improvement in balance abilities after strengthening exercises, previous studies showed that they are able to improve balance recovery and dynamic and static balance skills in older adults, and consequently prevent falls (72, 73), whereas for others, the use of strengthening exercises alone failed to show clear effects on balance abilities (68, 74). One RCT in this review found that resistance exercises when done on either stable or unstable surfaces using exercise machines or free weights, demonstrated significant improvements in muscle power and reactive balance, with no significant differences between the three interventions, though gains in muscle strength (isometric leg extension strength) and gains in muscle power (chair rise test) were greater in exercises accomplished on unstable surfaces. Therefore, the authors recommended following resistance training with unstable surfaces and moderate instability as in machine-based unstable resistance training if the goal is to enhance muscle strength and the power of the lower extremities. While if great load is a concern and limited to a degree, then free-weight unstable resistance training is recommended (52).

In the same way as strength training, balance training when followed as a single intervention revealed no evidence to be effective in reducing the rate of falls and the risk of falling (23), yet the inclusion of balance training in exercise programs appeared to be a crucial factor in reducing falls (23, 27, 35, 37). Additionally, it is recommended that exercises must provide a moderate or high challenge to balance in order to prevent falls (37). Hence, this explained that different exercise programs that included balance training were similarly effective in reducing falls (35, 75, 76). According to one RCT in our review, a higher effect of balance intervention in reducing falls can be found in studies that are of a single-center nature (42). A previous study found that balance training on a foam rubber affected postural reflex in older adults (77) through improvement in the proprioception and sensitivity of lower limbs and cutaneous receptors in the soles (78). An interesting result in our review is that both foam rubber and stable surface interventions improved lower extremity muscle strength in addition to balance, with no significant difference between both (43). This is consistent with other studies that reported improvement in muscle strength after balance interventions (33, 34, 36). Other studies found a relationship between single-leg standing similar to the intervention in this trial and muscle activation (79, 80). This would prove that the progressive step marching exercise in our review is able to improve lower limb strength and eventually reduce the rate of falls in older adults (62). On the contrary, one trial we investigated did not reveal significant improvement in leg strength outcomes at the end of the balance intervention compared with pre-intervention, however, it was significant when compared to a combined intervention of virtual balance and static and dynamic balance exercises (60).

Perturbation-based balance exercises showed improvement in local dynamic stability and balance recovery with effects lasting for a week after the intervention, though it was a one-session intervention (47), others showed a reduction in the rate of all falls and slip falls (57). This is in agreement with other studies that showed quick effects (81, 82) and long-lasting ones with marginal reduction in gains (83, 84). Other trials of perturbation training in this review with longer intervention exhibited significant improvement in balance confidence using the ABC scale, dynamic stability, balance ability, and functional capacity compared to control, even more effective than traditional balance training (50, 53). Mean scores on the ABC scale improved from 65 to 80. One study revealed that the ABC scale is a strong predictor of falls in older adults, with those with a score < 67 having a high probability to fall (82). Others reported improvement in balance confidence after 4 weeks of perturbation training (85).

The multi-component intervention included a combination of trunk stability exercises and stretching (63), demonstrating significant improvement in complex walking ability. Combined strength and aerobic exercise interventions led to enhancement of lower limb strength (58), and improvement in aerobic capacity (63). In a study, it was found that improvements in balance and mobility persisted for up to 1 year after participating in a-32 week combined resistance and flexibility training program (86). Others reported that multi-component interventions had positive effects on the rate of falls (23, 27, 87, 88) and the risk of falling (23, 27) in addition to improvements in strength and balance outcomes (87, 88). Multiple categories of exercise programs containing balance training and muscle strengthening demonstrated their effectiveness in reducing both rate of falls and the risk of falling (23), whereas for others, balance and functional exercises plus strengthening revealed the same effectiveness (27). A previous study of combined strengthening and balance interventions found that physical performance was maintained during the follow-up, and that medically-attended injurious falls were significantly less after 5 years, while gains diminished in strengthening-only or balance-only interventions (89). It has been recommended that exercises for fall prevention must be of sufficient doses (i.e., at least 2 h a week) as well as in an ongoing manner (37).

Chronic diseases and multimorbidity (i.e., the coexistence of ≥2 chronic diseases in the same person) are prevalent among adults over the age of 65 years (90–93), and studies found that coronary artery diseases, stroke, diabetes, and arthritis are associated with physical inactivity in the older population (94, 95). However, participating in physical activity is believed to prevent and reduce the number of chronic diseases in older adults. (96). Others reported that the risks of falls and recurrent falling are directly proportional to the number of chronic diseases (97, 98). Frailty (99) and polypharmacy (100) (i.e., taking ≥2 drugs) are possible explanations for the connections with multimorbidity.

When studying the multimorbidity patterns (i.e., the classification of chronic diseases into different combinations based on the associations between them), the increased risk of falls was higher in the visceral-arthritic and mental-sensory patterns (97), while recurrent falling was significantly associated with a cluster of the highest prevalence of osteoporosis and a cluster that had the highest number of chronic conditions (98).

Therefore, practicing regular physical activity is crucial in reducing and preventing chronic diseases, maintaining a healthy musculoskeletal system and balance, and reducing the risk of falling.

A major strength of our review is that, to our knowledge, it is the first systematic review of RCTs to investigate the effects of different exercise interventions on ambulatory community-dwelling older adults. This study has several limitations that need to be taken into consideration. First, this review was limited to older adults who were free of dementia, stroke, Parkinson’s disease, multiple sclerosis, or recent fractures, therefore findings cannot be generalized to higher-risk populations. Second, trials with home exercise programs and virtual reality exercises were excluded. Third, the review results can only be generalized to home-dwelling older adults; trials with institutionalized older adults were excluded as well. Fourth, performing a meta-analysis was not possible due to the heterogeneity available in the included studies; variabilities in outcomes and interventions that obviously appear in prior sections of the current review. Therefore, as an alternative to a meta-analysis, we used other methods to compare the effects among the included studies. Finally, there is a lack of RCTs that particularly consider older adults with limited access to health care or in low-income countries.

## 5. Conclusion

In conclusion, our review addressed numerous RCTs of physical exercises composed of strengthening, balance, or multi-component interventions on physical functionality and risk of falls in community-dwelling older adults. The present review confirms the previous findings that physical exercise improves the components of functional ability, i.e., lower extremity strength, balance, and mobility. Additionally, physical exercise reduces falls and minimizes their serious sequelae. Further research on fall prevention is required that specifically considers high-risk older adults, low-income countries, and older adults in vulnerable contexts with limited access to health care.

## Data availability statement

The original contributions presented in the study are included in the article/Supplementary material, further inquiries can be directed to the corresponding author.

## Author contributions

MS: conceptualization, methodology, investigation, writing—original draft, and supervision. ZN: methodology and investigation. AM: proofreading and editing. VP: proofreading, editing, administrative support, and coordination. MH: conceptualization, methodology, proofreading, editing, and supervision. All authors contributed to the article and approved the submitted version.

## Funding

This research was funded by the Thematic Excellence Program 2020—Institutional Excellence Sub-program/National Excellence Sub-program of the Ministry for Innovation and Technology in Hungary, within the framework of the 3. thematic program of the University of Pécs (TKP2021-EGA-10) and supported by the ÚNKP-22-4-II-PTE-1667 New National Excellence Program of the Ministry for Innovation and Technology from the source of the National Research, Development, and Innovation Fund. The authors declare that the funding sources did not have any role in the study design; in the collection, analysis, and interpretation of the data, or in writing and submitting this manuscript.

## Conflict of interest

The authors declare that the research was conducted in the absence of any commercial or financial relationships that could be construed as a potential conflict of interest.

## Publisher’s note

All claims expressed in this article are solely those of the authors and do not necessarily represent those of their affiliated organizations, or those of the publisher, the editors and the reviewers. Any product that may be evaluated in this article, or claim that may be made by its manufacturer, is not guaranteed or endorsed by the publisher.

## Supplementary material

The Supplementary material for this article can be found online at: https://www.frontiersin.org/articles/10.3389/fpubh.2023.1209319/full#supplementary-material

Supplementary Figure S1 Risk of bias summary.

Supplementary Figure S2 Risk of bias graph.

## Abbreviations

2MST: 2-minutes step test
ABC: Activities-specific balance confidence
CST: Chair standing test
F8WT: Figure-of-8 walking test
FSST: Four square step test
MME: Multimodal exercise
RCT: Randomized controlled trial
SE: Stretching exercises
SLST: Single leg stance test
TUGT: Timed up and go test
WHO: World Health Organization

## References

[1] Tinetti ME. Preventing falls in elderly persons. N Engl J Med. (2003) 348:42–9. doi: 10.1056/NEJMcp020719 12510042

[2] Kalache A Fu D Yoshida S Al-Faisal W Beattie L Chodzko-Zajko W. World Health Organisation global report on falls prevention in older age. Geneva: World Health Organization (2007). Available at: http://www.who.int/ageing/publications/Falls_prevention7March.pdf (Accessed March 10, 2021).

[3] Peeters G van Schoor NM Lips P. Fall risk: the clinical relevance of falls and how to integrate fall risk with fracture risk. Best Pract Res Clin Rheumatol. (2009) 23:797–804. doi: 10.1016/j.berh.2009.09.004, PMID: 19945691

[4] Turner S Kisser R Rogmans W. Fall among older adults in the EU-28. The European Public Health Association. (2015) Available at: https://eupha.org/repository/sections/ipsp/Factsheet_falls_in_older_adults_in_EU.pdf (Accessed March 10, 2021).

[5] Uusi-Rasi K Patil R Karinkanta S Kannus P Tokola K Lamberg-Allardt C. A 2-year follow-up after a 2-year RCT with vitamin D and exercise: effects on falls, injurious falls and physical functioning among older women. J Gerontol Ser A. (2017) 72:1239–45. doi: 10.1093/gerona/glx044, PMID: 28369286 PMC5861967

[6] Deandrea S Lucenteforte E Bravi F Foschi R La Vecchia C Negri E. Risk factors for falls in community-dwelling older people: a systematic review and Meta-analysis. Epidemiology. (2010) 21:658–68. doi: 10.1097/EDE.0b013e3181e89905 20585256

[7] Karinkanta S Piirtola M Sievänen H Uusi-Rasi K Kannus P. Physical therapy approaches to reduce fall and fracture risk among older adults. Nat Rev Endocrinol. (2010) 6:396–407. doi: 10.1038/nrendo.2010.70, PMID: 20517287

[8] Lee TW Ko IS Lee KJ. Health promotion behaviors and quality of life among community-dwelling elderly in Korea: a cross-sectional survey. Int J Nurs Stud. (2006) 43:293–300. doi: 10.1016/j.ijnurstu.2005.06.009, PMID: 16105668

[9] Benichou O Lord SR. Rationale for strengthening muscle to prevent falls and fractures: a review of the evidence. Calcif Tissue Int. (2016) 98:531–45. doi: 10.1007/s00223-016-0107-9, PMID: 26847435

[10] Toraman A Yıldırım NÜ. The falling risk and physical fitness in older people. Arch Gerontol Geriatr. (2010) 51:222–6. doi: 10.1016/j.archger.2009.10.012 19939475

[11] Boyé ND Van Lieshout EM Van Beeck EF Hartholt KA Van der Cammen TJ Patka P. The impact of falls in the elderly. Trauma. (2013) 15:29–35. doi: 10.1177/1460408612463145

[12] Franco MR Pereira LS Ferreira PH. Exercise interventions for preventing falls in older people living in the community. Br J Sports Med. (2014) 48:867–8. doi: 10.1136/bjsports-2012-092065 23314888

[13] Paterson DH Warburton DE. Physical activity and functional limitations in older adults: a systematic review related to Canada’s physical activity guidelines. Int J Behav Nutr Phys Act. (2010) 7:38. doi: 10.1186/1479-5868-7-38, PMID: 20459782 PMC2882898

[14] Wang RY Wang YL Cheng FY Chao YH Chen CL Yang YR. Effects of combined exercise on gait variability in community-dwelling older adults. Age. (2015) 37:9780. doi: 10.1007/s11357-015-9780-2, PMID: 25907712 PMC4408301

[15] Ács P Stocker M Füge K Paár D Oláh A Kovács A. Economic and public health benefits: the result of increased regular physical activity. Eur J Integr Med. (2016) 8:8–12. doi: 10.1016/j.eujim.2016.11.003

[16] Petersen RC Smith GE Waring SC Ivnik RJ Tangalos EG Kokmen E. Mild cognitive impairment: clinical characterization and outcome. Arch Neurol. (1999) 56:303–8. doi: 10.1001/archneur.56.3.303 10190820

[17] Lara E Koyanagi A Caballero F Domènech-Abella J Miret M Olaya B. Cognitive reserve is associated with quality of life: a population-based study. Exp Gerontol. (2017) 87:67–73. doi: 10.1016/j.exger.2016.10.012, PMID: 27825839

[18] Sánchez-González JL Calvo-Arenillas JI Sánchez-Rodríguez JL. The effects of moderate physical exercise on cognition in adults over 60 years of age. Rev Neurol. (2018) 66:230–6. doi: 10.33588/rn.6607.2017449, PMID: 29557548

[19] Sánchez-González JL Sánchez-Rodríguez JL Martín-Vallejo J Martel-Martel A González-Sarmiento R. Effects of physical exercise on cognition and telomere length in healthy older women. Brain Sci. (2021) 11:1417. doi: 10.3390/brainsci11111417, PMID: 34827416 PMC8615568

[20] Boente-Antela B Leirós-Rodríguez R García-Soidán JL. Compliance with the recommendations of the World Health Organization on the practice of physical activity in people over 65 years in Spain. J Hum Sport Exerc. (2020) 17:29–38. doi: 10.14198/jhse.2022.171.04

[21] World Health Organization. Global recommendations on physical activity for health (2010). Available at: https://www.who.int/publications/i/item/9789241599979 (Accessed March 14, 2021). 26180873

[22] World Health Organization. WHO global report on falls prevention in older age. (2008). Available at: https://apps.who.int/iris/handle/10665/43811 (Accessed March 14, 2021).

[23] Gillespie LD Robertson MC Gillespie WJ Sherrington C Gates S Clemson L. Interventions for preventing falls in older people living in the community. Cochrane Database Syst Rev. (2012) 2021:CD007146. doi: 10.1002/14651858.CD007146.pub3, PMID: 22972103 PMC8095069

[24] Paillard T Lafont C Costes-Salon MC Rivière D Dupui P. Effects of brisk walking on static and dynamic balance, locomotion, body composition, and aerobic capacity in ageing healthy active men. Int J Sports Med. (2004) 25:539–46. doi: 10.1055/s-2004-820948, PMID: 15459836

[25] Palvanen M Kannus P Piirtola M Niemi S Parkkari J Järvinen M. Effectiveness of the Chaos falls clinic in preventing falls and injuries of home-dwelling older adults: a randomised controlled trial. Injury. (2014) 45:265–71. doi: 10.1016/j.injury.2013.03.010 23579066

[26] Sherrington C Michaleff ZA Fairhall N Paul SS Tiedemann A Whitney J. Exercise to prevent falls in older adults: an updated systematic review and meta-analysis. Br J Sports Med. (2017) 51:1750–8. doi: 10.1136/bjsports-2016-096547 27707740

[27] Sherrington C Fairhall NJ Wallbank GK Tiedemann A Michaleff ZA Howard K. Exercise for preventing falls in older people living in the community. Cochrane Database Syst Rev. (2019) 2019:CD012424. doi: 10.1002/14651858.CD012424.pub2, PMID: 30703272 PMC6360922

[28] Shimada H Obuchi S Furuna T Suzuki T. New intervention program for preventing falls among frail elderly people: the effects of perturbed walking exercise using a bilateral separated treadmill. Am J Phys Med Rehabil. (2004) 83:493–9. doi: 10.1097/01.PHM.0000130025.54168.91 15213472

[29] Korpelainen R Keinänen-Kiukaanniemi S Nieminen P Heikkinen J Väänänen K Korpelainen J. Long-term outcomes of exercise: follow-up of a randomized trial in older women with osteopenia. Arch Intern Med. (2010) 170:1548–56. doi: 10.1001/archinternmed.2010.311 20876406

[30] Panel on Prevention of Falls in Older Persons, American Geriatrics Society, and British geriatrics society. Summary of the updated American Geriatrics Society/British geriatrics society clinical practice guideline for prevention of falls in older persons. J Am Geriatr Soc. (2011) 59:148–57. doi: 10.1111/j.1532-5415.2010.03234.x 21226685

[31] Grgic J Garofolini A Orazem J Sabol F Schoenfeld BJ Pedisic Z. Effects of resistance training on muscle size and strength in very elderly adults: a systematic review and meta-analysis of randomized controlled trials. Sports Med. (2020) 50:1983–99. doi: 10.1007/s40279-020-01331-7, PMID: 32740889

[32] LaStayo P Marcus R Dibble L Wong B Pepper G. Eccentric versus traditional resistance exercise for older adult fallers in the community: a randomized trial within a multi-component fall reduction program. BMC Geriatr. (2017) 17:149. doi: 10.1186/s12877-017-0539-8, PMID: 28716003 PMC5513167

[33] Audette JF Jin YS Newcomer R Stein L Duncan G Frontera WR. Tai Chi versus brisk walking in elderly women. Age Ageing. (2006) 35:388–93. doi: 10.1093/ageing/afl006, PMID: 16624847

[34] Heitkamp HC Horstmann T Mayer F Weller J Dickhuth HH. Gain in strength and muscular balance after balance training. Int J Sports Med. (2001) 22:285–90. doi: 10.1055/s-2001-13819, PMID: 11414672

[35] Sherrington C Whitney JC Lord SR Herbert RD Cumming RG Close JCT. Effective exercise for the prevention of falls: a systematic review and Meta-analysis. J Am Geriatr Soc. (2008) 56:2234–43. doi: 10.1111/j.1532-5415.2008.02014.x, PMID: 19093923

[36] Taylor-Piliae RE Haskell WL Stotts NA Froelicher ES. Improvement in balance, strength, and flexibility after 12 weeks of tai chi exercise in ethnic Chinese adults with cardiovascular disease risk factors. Altern Ther Health Med. (2006) 12:50–8. doi: 10.1016/j.ejcnurse.2005.10.008, PMID: 16541997

[37] Sherrington C Tiedemann A Fairhall N Close JCT Lord SR. Exercise to prevent falls in older adults: an updated meta-analysis and best practice recommendations. N S W Public Health Bull. (2011) 22:78–83. doi: 10.1071/NB10056 21632004

[38] Higgins JPT Altman DG Gøtzsche PC Jüni P Moher D Oxman AD. The Cochrane Collaboration’s tool for assessing risk of bias in randomised trials. BMJ. (2011) 343:d5928. doi: 10.1136/bmj.d5928, PMID: 22008217 PMC3196245

[39] Arnold C Lanovaz J Oates A Craven B Butcher S. The effect of adding core stability training to a standard balance exercise program on sit to stand performance in older adults: a pilot study. J Aging Phys Act. (2015) 23:95–102. doi: 10.1123/JAPA.2013-0115, PMID: 24451365

[40] Li F Harmer P Fitzgerald K Eckstrom E Akers L Chou LS. Effectiveness of a therapeutic tai Ji Quan intervention vs a multimodal exercise intervention to prevent falls among older adults at high risk of falling: a randomized clinical trial. JAMA Intern Med. (2018) 178:1301–10. doi: 10.1001/jamainternmed.2018.3915, PMID: 30208396 PMC6233748

[41] Li F Harmer P Eckstrom E Fitzgerald K Chou LS Liu Y. Effectiveness of tai Ji Quan vs multimodal and stretching exercise interventions for reducing injurious falls in older adults at high risk of falling: follow-up analysis of a randomized clinical trial. JAMA Netw Open. (2019) 2:e188280. doi: 10.1001/jamanetworkopen.2018.8280, PMID: 30768195 PMC6484587

[42] El-Khoury F Cassou B Latouche A Aegerter P Charles MA Dargent-Molina P. Effectiveness of two year balance training programme on prevention of fall induced injuries in at risk women aged 75-85 living in community: Ossébo randomised controlled trial. BMJ. (2015) 351:h3830. doi: 10.1136/bmj.h3830, PMID: 26201510 PMC4511529

[43] Hirase T Inokuchi S Matsusaka N Okita M. Effects of a balance training program using a foam rubber pad in community-based older adults: a randomized controlled trial. J Geriatr Phys Ther. (2015) 38:62–70. doi: 10.1519/JPT.0000000000000023, PMID: 24978931

[44] Lacroix A Kressig RW Muehlbauer T Gschwind YJ Pfenninger B Bruegger O. Effects of a supervised versus an unsupervised combined balance and strength training program on balance and muscle power in healthy older adults: a randomized controlled trial. Gerontology. (2016) 62:275–88. doi: 10.1159/000442087, PMID: 26645282

[45] Patil R Uusi-Rasi K Tokola K Karinkanta S Kannus P Sievänen H. Effects of a multimodal exercise program on physical function, falls, and injuries in older women: a 2-year community-based, randomized controlled trial. J Am Geriatr Soc. (2015) 63:1306–13. doi: 10.1111/jgs.13489, PMID: 26115073

[46] Johnson SL Stevens SL Fuller DK Caputo JL. Effect of lower-extremity eccentric training on physical function in community-dwelling older adults. Phys Occup Ther Geriatr. (2019) 37:298–312. doi: 10.1080/02703181.2019.1648626

[47] Rieger MM Papegaaij S Pijnappels M Steenbrink F van Dieën JH. Transfer and retention effects of gait training with anterior-posterior perturbations to postural responses after medio-lateral gait perturbations in older adults. Clin Biomech. (2020) 75:104988. doi: 10.1016/j.clinbiomech.2020.104988, PMID: 32174482

[48] Kocaman AA Kırdı N Aksoy S Elmas Ö Dogu BB. The effect of different exercise training types on functionality in older fallers: a pilot randomized controlled trial. Top Geriatr Rehabil. (2021) 37:114–27. doi: 10.1097/TGR.0000000000000312

[49] Ansai JH Aurichio TR Gonçalves R Rebelatto JR. Effects of two physical exercise protocols on physical performance related to falls in the oldest old: a randomized controlled trial: exercises protocols in oldest old. Geriatr Gerontol Int. (2016) 16:492–9. doi: 10.1111/ggi.12497, PMID: 25868484

[50] Arghavani H Zolaktaf V Lenjannejadian S. Comparing the effects of anticipatory postural adjustments focused training and balance training on postural preparation, balance confidence and quality of life in elderly with history of a fall. Aging Clin Exp Res. (2020) 32:1757–65. doi: 10.1007/s40520-019-01358-5, PMID: 31608424

[51] Chittrakul J Siviroj P Sungkarat S Sapbamrer R. Multi-system physical exercise intervention for fall prevention and quality of life in pre-frail older adults: a randomized controlled trial. Int J Environ Res Public Health. (2020) 17:3102. doi: 10.3390/ijerph17093102, PMID: 32365613 PMC7246743

[52] Eckardt N. Lower-extremity resistance training on unstable surfaces improves proxies of muscle strength, power and balance in healthy older adults: a randomised control trial. BMC Geriatr. (2016) 16:191. doi: 10.1186/s12877-016-0366-3, PMID: 27881086 PMC5122203

[53] Hamed A Bohm S Mersmann F Arampatzis A. Exercises of dynamic stability under unstable conditions increase muscle strength and balance ability in the elderly. Scand J Med Sci Sports. (2018) 28:961–71. doi: 10.1111/sms.13019, PMID: 29154407

[54] Jang EM Park SH. Effects of neuromuscular electrical stimulation combined with exercises versus an exercise program on the physical characteristics and functions of the elderly: a randomized controlled trial. Int J Environ Res Public Health. (2021) 18:2463. doi: 10.3390/ijerph18052463, PMID: 33802260 PMC7967594

[55] Karinkanta S Kannus P Uusi-Rasi K Heinonen A Sievänen H. Combined resistance and balance-jumping exercise reduces older women’s injurious falls and fractures: 5-year follow-up study. Age Ageing. (2015) 44:784–9. doi: 10.1093/ageing/afv064, PMID: 25990940

[56] Lee SH Lee DY. Effects of open and closed kinetic chain exercises on the balance using elastic bands for the health Care of the Elderly Females. Medico-Leg Update. (2019) 19:728. doi: 10.5958/0974-1283.2019.00263.9

[57] Okubo Y Sturnieks DL Brodie MA Duran L Lord SR. Effect of reactive balance training involving repeated slips and trips on balance recovery among older adults: a blinded randomized controlled trial. J Gerontol Ser A (2019) 16;74:1489–1496. doi: 10.1093/gerona/glz021 30721985

[58] Park J Lee J Yang J Lee B Han D. Effects of combined exercise on changes of lower extremity muscle activation during walking in older women. J Phys Ther Sci. (2015) 27:1515–8. doi: 10.1589/jpts.27.1515, PMID: 26157253 PMC4483431

[59] Pourtaghi F Moghadam ZE Ramezani M Vashani HB Mohajer S. The effect of resistance training using Thera-band on muscular strength and quality of life among the elderly in the City of Mashhad. Evid Based Care. (2017). 7:7–16. doi: 10.22038/ebcj.2017.25876.1584

[60] Sadeghi H Jehu DA Daneshjoo A Shakoor E Razeghi M Amani A. Effects of 8 weeks of balance training, virtual reality training, and combined exercise on lower limb muscle strength, balance, and functional mobility among older men: a randomized controlled trial. Sports Health Multidiscip Approach. (2021) 13:606–12. doi: 10.1177/1941738120986803, PMID: 33583253 PMC8558995

[61] Sannicandro I. Effects of strength and core stability training versus strength and aerobic training in subjects aged over 65. Med Sport. (2018) 70:410–8. doi: 10.23736/S0025-7826.17.03132-5

[62] Sitthiracha P Eungpinichpong W Chatchawan U. Effect of progressive step marching exercise on balance ability in the elderly: a cluster randomized clinical trial. Int J Environ Res Public Health. (2021) 18:3146. doi: 10.3390/ijerph18063146, PMID: 33803720 PMC8003065

[63] Song HS Kim JY. The effects of complex exercise on walking ability during direction change and falls efficacy in the elderly. J Phys Ther Sci. (2015) 27:1365–7. doi: 10.1589/jpts.27.1365, PMID: 26157220 PMC4483398

[64] Zhao Y Chung PK Tong TK. Effectiveness of a community-based exercise program on balance performance and fear of falling in older nonfallers at risk for falling: a randomized. Control Study J Aging Phys Act. (2016) 24:516–24. doi: 10.1123/japa.2015-0224, PMID: 26796916

[65] Zhao Y Chung PK Tong TK. Effectiveness of a balance-focused exercise program for enhancing functional fitness of older adults at risk of falling: a randomised controlled trial. Geriatr Nur. (2017) 38:491–7. doi: 10.1016/j.gerinurse.2017.02.011, PMID: 28359614

[66] Moreland JD Richardson JA Goldsmith CH Clase CM. Muscle weakness and falls in older adults: a systematic review and meta-analysis. J Am Geriatr Soc. (2004) 52:1121–9. doi: 10.1111/j.1532-5415.2004.52310.x 15209650

[67] American College of Sports Medicine Chodzko-Zajko WJ Proctor DN Fiatarone Singh MA Minson CT Nigg CR. American College of Sports Medicine position stand. Exercise and physical activity for older adults. Med Sci Sports Exerc. (2009) 41:1510–30. doi: 10.1249/MSS.0b013e3181a0c95c, PMID: 19516148

[68] Latham N Anderson C Bennett D Stretton C. Progressive resistance strength training for physical disability in older people. Cochrane Database Syst Rev. (2003) 2003:CD002759. doi: 10.1002/14651858.CD002759 12804434

[69] Singh NA Stavrinos TM Scarbek Y Galambos G Liber C Fiatarone Singh MA. A randomized controlled trial of high versus low intensity weight training versus general practitioner care for clinical depression in older adults. J Gerontol A Biol Sci Med Sci. (2005) 60:768–76. doi: 10.1093/gerona/60.6.768, PMID: 15983181

[70] Martins WR de Oliveira RJ Carvalho RS de Oliveira DV da Silva VZM Silva MS. Elastic resistance training to increase muscle strength in elderly: a systematic review with meta-analysis. Arch Gerontol Geriatr. (2013) 57:8–15. doi: 10.1016/j.archger.2013.03.002, PMID: 23562413

[71] LaStayo PC Ewy GA Pierotti DD Johns RK Lindstedt S. The positive effects of negative work: increased muscle strength and decreased fall risk in a frail elderly population. J Gerontol Ser A. (2003) 58:M419–24. doi: 10.1093/gerona/58.5.M419, PMID: 12730250

[72] Arampatzis A Peper A Bierbaum S. Exercise of mechanisms for dynamic stability control increases stability performance in the elderly. J Biomech. (2011) 44:52–8. doi: 10.1016/j.jbiomech.2010.08.023, PMID: 20832803

[73] Marques EA Figueiredo P Harris TB Wanderley FA Carvalho J. Are resistance and aerobic exercise training equally effective at improving knee muscle strength and balance in older women? Arch Gerontol Geriatr. (2017) 68:106–12. doi: 10.1016/j.archger.2016.10.002, PMID: 27764726 PMC5760177

[74] Orr R Raymond J Fiatarone SM. Efficacy of progressive resistance training on balance performance in older adults: a systematic review of randomized controlled trials. Sports Med Auckl NZ. (2008) 38:317–43. doi: 10.2165/00007256-200838040-00004 18348591

[75] Li F Harmer P Fisher KJ McAuley E Chaumeton N Eckstrom E. Tai chi and fall reductions in older adults: a randomized controlled trial. J Gerontol A Biol Sci Med Sci. (2005) 60:187–94. doi: 10.1093/gerona/60.2.187 15814861

[76] Robertson MC Campbell AJ Gardner MM Devlin N. Preventing injuries in older people by preventing falls: a meta-analysis of individual-level data. J Am Geriatr Soc. (2002) 50:905–11. doi: 10.1046/j.1532-5415.2002.50218.x 12028179

[77] Granacher U Gollhofer A Strass D. Training induced adaptations in characteristics of postural reflexes in elderly men. Gait Posture. (2006) 24:459–66. doi: 10.1016/j.gaitpost.2005.12.007, PMID: 16472525

[78] McIlroy WE Bishop DC Staines WR Nelson AJ Maki BE Brooke JD. Modulation of afferent inflow during the control of balancing tasks using the lower limbs. Brain Res. (2003) 961:73–80. doi: 10.1016/S0006-8993(02)03845-3, PMID: 12535778

[79] Dingenen B Janssens L Claes S Bellemans J Staes FF. Lower extremity muscle activation onset times during the transition from double-leg stance to single-leg stance in anterior cruciate ligament reconstructed subjects. Clin Biomech. (2016) 35:116–23. doi: 10.1016/j.clinbiomech.2016.04.014 27149566

[80] Iverson BD Gossman MR Shaddeau SA Turner ME Jr. Balance performance, force production, and activity levels in noninstitutionalized men 60 to 90 years of age. Phys Ther. (1990) 70:348–55. doi: 10.1093/ptj/70.6.348, PMID: 2345778

[81] Okubo Y Schoene D Lord SR. Step training improves reaction time, gait and balance and reduces falls in older people: a systematic review and meta-analysis. Br J Sports Med. (2017) 51:586–93. doi: 10.1136/bjsports-2015-095452, PMID: 26746905

[82] Pai YC Bhatt T Wang E Espy D Pavol MJ. Inoculation against falls: rapid adaptation by young and older adults to slips during daily activities. Arch Phys Med Rehabil. (2010) 91:452–9. doi: 10.1016/j.apmr.2009.10.032, PMID: 20298839 PMC2842602

[83] Parijat P Lockhart TE. Effects of moveable platform training in preventing slip-induced falls in older adults. Ann Biomed Eng. (2012) 40:1111–21. doi: 10.1007/s10439-011-0477-0, PMID: 22134467 PMC3319506

[84] Tanvi B Feng Y Yi-Chung P. Learning to resist gait-slip falls: long-term retention in community-dwelling older adults. Arch Phys Med Rehabil. (2012) 93:557–64. doi: 10.1016/j.apmr.2011.10.027, PMID: 22341989 PMC3667400

[85] Pijnappels M Reeves ND Maganaris CN van Dieën JH. Tripping without falling; lower limb strength, a limitation for balance recovery and a target for training in the elderly. J Electromyogr Kinesiol. (2008) 18:188–96. doi: 10.1016/j.jelekin.2007.06.004 17761436

[86] Bird M Hill KD Ball M Hetherington S Williams AD. The long-term benefits of a multi-component exercise intervention to balance and mobility in healthy older adults. Arch Gerontol Geriatr. (2011) 52:211–6. doi: 10.1016/j.archger.2010.03.021, PMID: 20416959

[87] Clemson L Munro J Singh MF. Lifestyle-integrated functional exercise (LiFE) program to prevent falls [Participant’s manual]. Sydney University Press (2014). Available at: https://www.jstor.org/stable/j.ctv176kt7j (Accessed March 14, 2021).

[88] Taguchi N Higaki Y Inoue S Kimura H Tanaka K. Effects of a 12-month multicomponent exercise program on physical performance, daily physical activity, and quality of life in very elderly people with minor disabilities: an intervention study. J Epidemiol. (2010) 20:21–9. doi: 10.2188/jea.je20081033, PMID: 19897943 PMC3900776

[89] Karinkanta S Heinonen A Sievänen H Uusi-Rasi K Fogelholm M Kannus P. Maintenance of exercise-induced benefits in physical functioning and bone among elderly women. Osteoporos Int. (2009) 20:665–74. doi: 10.1007/s00198-008-0703-2, PMID: 18696173

[90] Thorpe KE Howard DH. The rise in spending among Medicare beneficiaries: the role of chronic disease prevalence and changes in treatment intensity. Health Aff. (2006) 25:w378–88. doi: 10.1377/hlthaff.25.w378 16926180

[91] Freedman VA Martin LG. Contribution of chronic conditions to aggregate changes in old-age functioning. Am J Public Health. (2000) 90:1755–60. doi: 10.2105/ajph.90.11.1755, PMID: 11076245 PMC1446390

[92] Wolff JL Starfield B Anderson G. Prevalence, expenditures, and complications of multiple chronic conditions in the elderly. Arch Intern Med. (2002) 162:2269–76. doi: 10.1001/archinte.162.20.2269, PMID: 12418941

[93] Vogeli C Shields AE Lee TA Gibson TB Marder WD Weiss KB. Multiple chronic conditions: prevalence, health consequences, and implications for quality, care management, and costs. J Gen Intern Med. (2007) 22 Suppl 3:391–5. doi: 10.1007/s11606-007-0322-1, PMID: 18026807 PMC2150598

[94] Kruger J Ham SA Sanker S. Physical inactivity during leisure time among older adults--behavioral risk factor surveillance system, 2005. J Aging Phys Act. (2008) 16:280–91. doi: 10.1123/japa.16.3.280 18660551

[95] Kumthekar A Pedro S Michaud K Ozen G Katz P Baker J. Physical activity habits among older adults living with rheumatic disease. J Rheumatol. (2023) 50:835–41. doi: 10.3899/jrheum.211244, PMID: 36642435

[96] Marques A Peralta M Martins J de Matos MG Brownson RC. Cross-sectional and prospective relationship between physical activity and chronic diseases in European older adults. Int J Public Health. (2017) 62:495–502. doi: 10.1007/s00038-016-0919-4, PMID: 27988796

[97] Yan J Wang M Cao Y. Patterns of multimorbidity in association with falls among the middle-aged and older adults: results from the China health and retirement longitudinal study. BMC Public Health. (2022) 22:1814. doi: 10.1186/s12889-022-14124-6, PMID: 36153523 PMC9508710

[98] Immonen M Haapea M Similä H Enwald H Keränen N Kangas M. Association between chronic diseases and falls among a sample of older people in Finland. BMC Geriatr. (2020) 20:225. doi: 10.1186/s12877-020-01621-9, PMID: 32590946 PMC7318483

[99] Abad-Díez JM Calderón-Larrañaga A Poncel-Falcó A Poblador-Plou B Calderón-Meza JM Sicras-Mainar A. Age and gender differences in the prevalence and patterns of multimorbidity in the older population. BMC Geriatr. (2014) 14:75. doi: 10.1186/1471-2318-14-75, PMID: 24934411 PMC4070347

[100] Fried TR O’Leary J Towle V Goldstein MK Trentalange M Martin DK. Health outcomes associated with polypharmacy in community-dwelling older adults: a systematic review. J Am Geriatr Soc. (2014) 62:2261–72. doi: 10.1111/jgs.13153, PMID: 25516023 PMC4270076

