# Supplementary figures and images for: Effectiveness of exercise interventions on fall prevention in ambulatory community-dwelling older adults: a systematic review with narrative synthesis
Source: Front Public Health. 2023 Aug 3;11:1209319. doi: 10.3389/fpubh.2023.1209319 (PMC10435089; doi:10.3389/fpubh.2023.1209319)

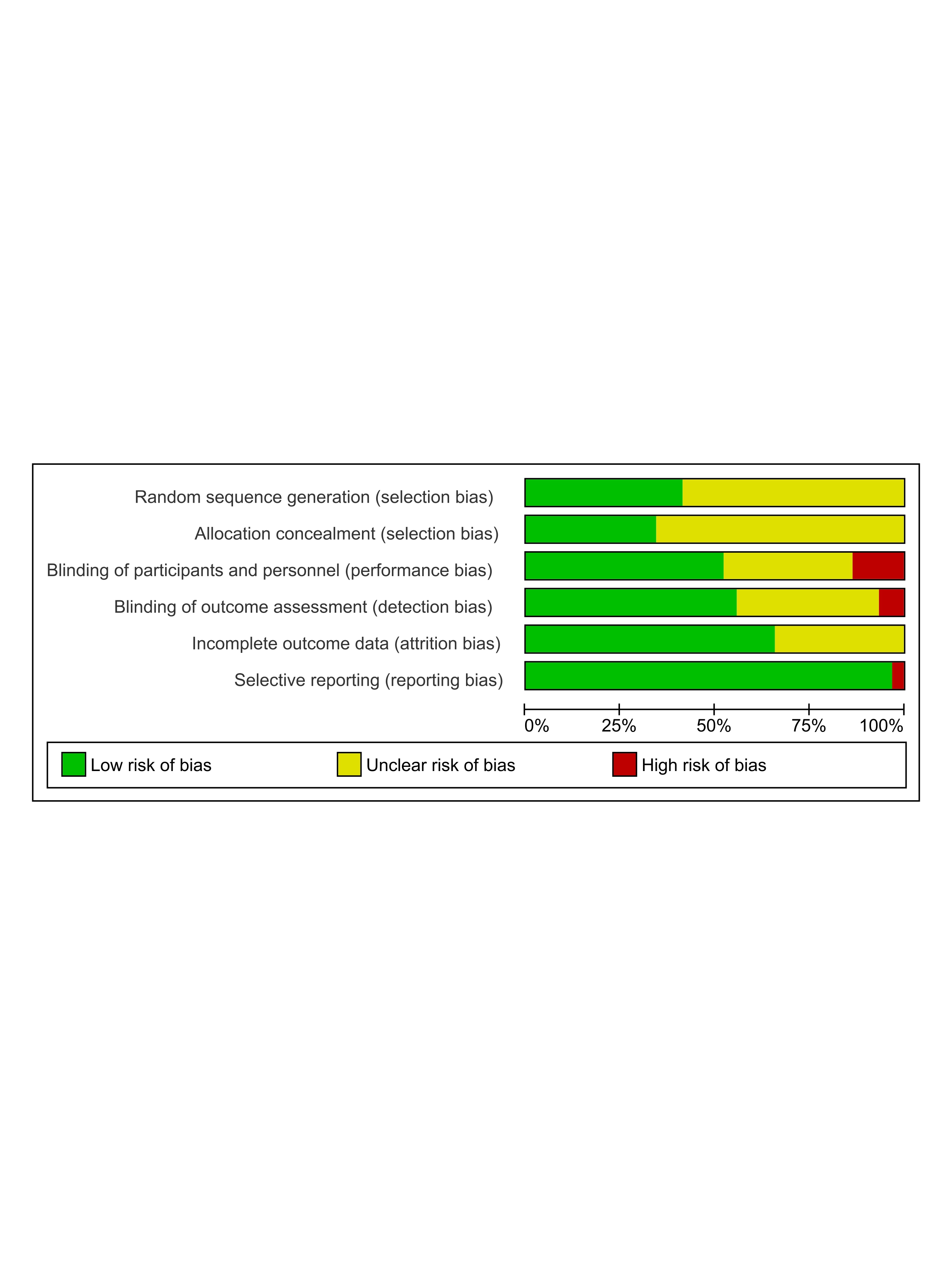

Supplement: Supplementary Figure S1 — Risk of bias summary. [file Image_1.JPEG]

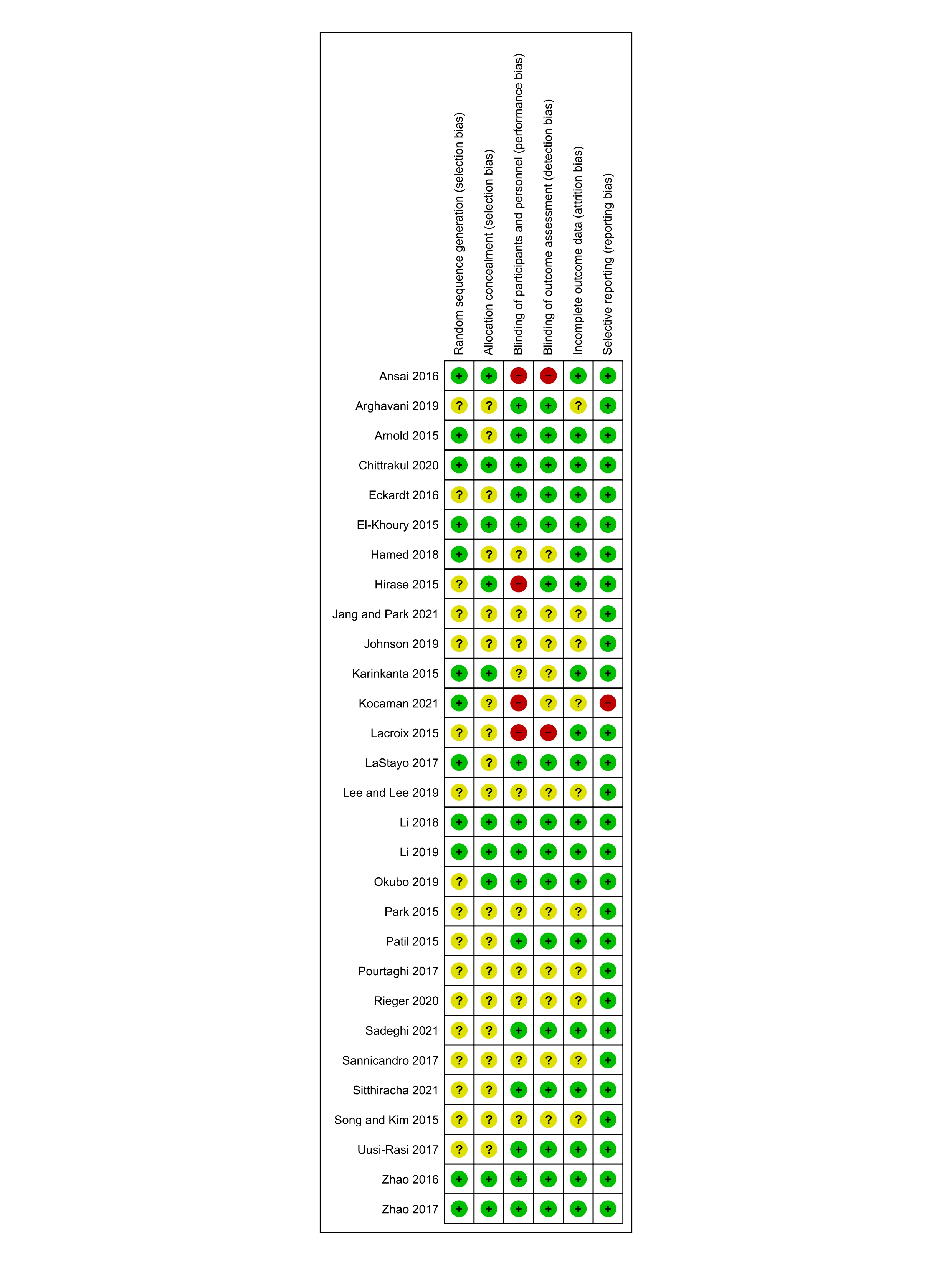

Supplement: Supplementary Figure S2 — Risk of bias graph. [file Image_2.JPEG]
